# Pig Weight and Body Size Estimation Using a Multiple Output Regression Convolutional Neural Network: A Fast and Fully Automatic Method

**DOI:** 10.3390/s21093218

**Published:** 2021-05-06

**Authors:** Jianlong Zhang, Yanrong Zhuang, Hengyi Ji, Guanghui Teng

**Affiliations:** 1 College of Water Resources & Civil Engineering, China Agricultural University, Beijing 100083, China; zhangjianlong@cau.edu.cn (J.Z.); zyr123@cau.edu.cn (Y.Z.); s20203091757@cau.edu.cn (H.J.); 2 Key Laboratory of Agricultural Engineering in Structure and Environment, Ministry of Agriculture and Rural Affairs, Beijing 100083, China; 3 Beijing Engineering Research Center on Animal Healthy Environment, Beijing 100083, China

**Keywords:** pig weight, body size, estimation, deep learning, convolutional neural network

## Abstract

Pig weight and body size are important indicators for producers. Due to the increasing scale of pig farms, it is increasingly difficult for farmers to quickly and automatically obtain pig weight and body size. Due to this problem, we focused on a multiple output regression convolutional neural network (CNN) to estimate pig weight and body size. DenseNet201, ResNet152 V2, Xception and MobileNet V2 were modified into multiple output regression CNNs and trained on modeling data. By comparing the estimated performance of each model on test data, modified Xception was selected as the optimal estimation model. Based on pig height, body shape, and contour, the mean absolute error (MAE) of the model to estimate body weight (BW), shoulder width (SW), shoulder height (SH), hip width (HW), hip width (HH), and body length (BL) were 1.16 kg, 0.33 cm, 1.23 cm, 0.38 cm, 0.66 cm, and 0.75 cm, respectively. The coefficient of determination (R^2^) value between the estimated and measured results was in the range of 0.9879–0.9973. Combined with the LabVIEW software development platform, this method can estimate pig weight and body size accurately, quickly, and automatically. This work contributes to the automatic management of pig farms.

## 1. Introduction

Animal husbandry is shifting toward automation, intelligence, and precision [1,2]. Pig weight and body size, two of the most important indicators for pig producers, provide information about feed conversion ratio (FCR), growth rate, uniformity, and health conditions [3,4]. Weight and body size also provide important references to regulate nutrition and the environment [5,6]. Precisely and automatically weighing pigs and measuring their body size can improve the feeding, breeding management, and selling, as well as preventing raisers from incurring unnecessary costs, humanpower, and materials, consequently improving the economic benefits [7,8].

Pig weight and body size are traditionally measured using ground scales and measuring sticks. This process causes stress to the animals and requires tremendous effort on behalf of the farm workers [9]. With the development of machine vision technology over the last 30 years, several researchers have searched for methods to estimate pig weight and body size using images to avoid direct measurements [10,11,12,13]. The estimation methods can be divided into four categories:

(i) Projection method. Project a slide with grids onto the back of a pig, then calculate the pig shoulder height and area according to the principle of stereo projection to estimate pig weight [14]. This method is difficult to automate.
(ii) Two-dimensional image method. Extract the pig body size, back area size, and other parameters from 2D images of pig backs, and use the model of the relationship between the pig weight and these parameters to achieve weight estimation. The average error of the weight estimation using this method is 3.38–5.3% [15,16,17,18].
(iii) Three-dimensional image method. After acquiring 3D images of pig backs using a depth camera, extract the pig back height, body size, back area size, and other parameters from the 3D image and use these parameters to estimate the pig weight. The 2D image mainly shows color, texture and contour information of the pig back, but the color and texture information are not related to the pig weight and body size. The 3D image shows outline and height information on the pig back; these parameters are highly correlated with the body size and pig weight. In addition, it was impossible to estimate the pig height using the 2D image. Therefore, this method is more promising than the 2D image method. The mean absolute error (MAE) of estimating pig body size for this method is 1.44–5.81% [9,19,20,21,22,23,24,25].
(iv) Ellipse fitting method. The ellipse fitting method is used to fit the area of a pig back image and estimate the weight of the pig based on the relationship model between the pig weight and center of mass, the length of the long axis and the short axis, the area, and the regional eccentricity of the fitted ellipse. The average relative error when using the ellipse fitting method to estimate pig weight is 3–3.8% [26,27,28,29].

In most of the aforementioned studies, the pig body images generally need to be processed as follows: background removal, image enhancement, image binarization, filtering and denoising, and head and tail removal, followed by the extraction of body size, volume, back area, and other parameters. The entire image process is cumbersome and time-consuming, and there is a chance of failure, all of which pose obstacles to automation.

The convolutional neural network (CNN) is one of the representative algorithms of deep learning. It is a type of feed-forward neural network that includes convolution calculation and has a deep structure. A CNN generally includes convolutional layers, pooling layers, fully connected layers, and an output layer, using the back propagation algorithm for the model training process [30]. Trained CNNs can extract information from images in an end-to-end manner with fast processing speed, and have been widely used in animal farming [31], clinical diagnosis [32], industrial production [33], and other aspects. In some equipment such as sorting systems for fattening pigs and breeding stations, there are strict requirements on the speed of pig weight and body size acquisition to improve operating efficiency. Due to the cumbersome and time-consuming process of the existing weight estimation methods and the real-time processing of images by CNN, a multiple output regression CNN model may be able to extract body shape features and estimate pig weight and body size quickly and accurately.

Given the above rationale, we aimed to develop a pig weight and body size estimation method using 3D images and a multiple output regression CNN. The study objectives were: (i) to train and select a pig weight and body size estimation model, (ii) to test the accuracy of this model, and (iii) to apply the method.

## 2. Materials and Methods

### 2.1. Design of the Pig Weight and Back Image Acquisition System

**Figure 1:**
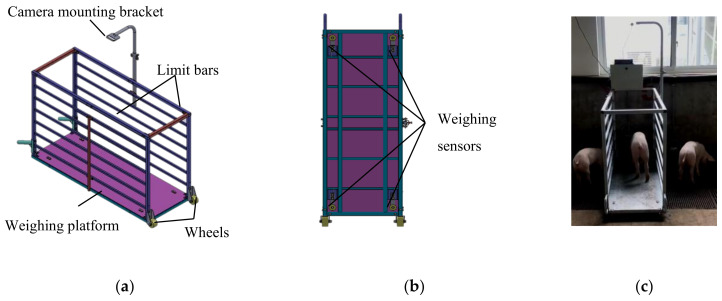
Pig weight and back image acquisition system: (**a**) three-dimensional diagram of system; (**b**) distribution of weighing sensors; (**c**) photo of system.

To train and evaluate the pig weight and body size estimation model, pig weight data, body size data, and 3D images of pig backs were needed. For pig weight data and 3D images of pig backs, the pig weight and back image acquisition system was designed (Figure 1). The size of the system is 1.5 mL × 0.5 mW × 0.9 mH. In the top of the system, there is an Intel RealSense D435 depth camera with a resolution of 1280 × 720 pixels to acquire 3D and 2D images simultaneously. There are 4 weighing sensors with a measurement range of 0–500 kg at the bottom of system, and the measurement accuracy after calibration was ±0.1 kg. The limit bars on both sides of the system ensure that the whole pig is on the scale when weighing. The acquisition system could easily move when necessary as it is on wheels.

**Figure 2:**
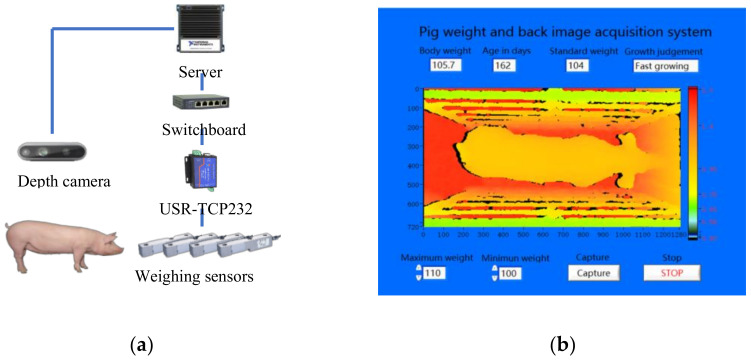
Pig weight and back image acquisition system: (**a**) data acquisition scheme; (**b**) software interface.

The system control program was developed based on the Internet of Things and the LabVIEW V18.0 software development platform, using Client-Server (C/S) architecture. The specific control scheme and program interface used are shown in Figure 2. The depth camera was connected to the server through a USB interface, and the pig weight data obtained by the weighing sensors were converted into a network signal by the USR-TCP232 and transmitted to the server through a switchboard. All software and hardware were controlled by the server, and MySQL version 5.5 database was installed in the server to store pig weight and image data. To ensure the quality of the acquired images, the running time of the system for this study was 8:00–17:00. When the program started, the depth camera and weighing sensors were initialized. Then, the system read the weighing data every 0.2 s. When 4 consecutive weighing data points were within the range of the pig population, and the difference between the maximum and minimum of the 4 data points was less than 0.2 kg, it was assumed that there was a pig on the weighing platform and that the pig was relatively quiet. When these requirements were met, the camera acquired the 3D and 2D images of the pig back simultaneously and the pig weight data and back images were saved in the database, so pig weight and back image data were able to be acquired continuously. Pig back images could also be manually acquired by using the capture button. The acquired 3D and 2D images were in PNG and APD format, respectively, and the file name of the 3D and 2D images was the acquisition time (accurate to milliseconds).

### 2.2. Acquisition Method of Body Size

**Figure 3:**
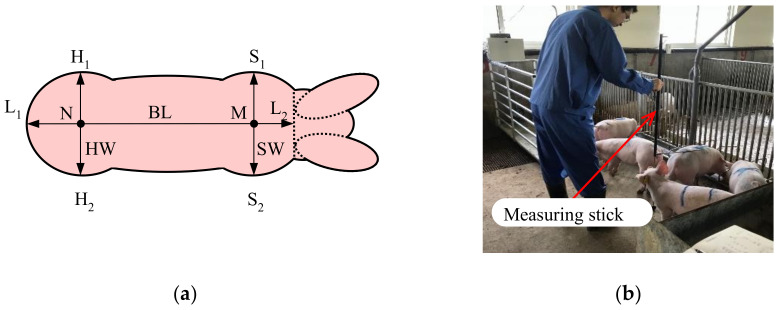
Specific locations of body size parameters and measurement of body size: (**a**) specific locations of body size parameters; (**b**) body size measurement using a measuring stick.

As shown in Figure 3a, the body size data includes body length (BL), shoulder width (SW), shoulder height (SH), hip width (HW), and hip height (HH). BL is the length of line L_1_–L_2_, which is the straight-line distance from the root of the ears to the root of the tail. The SW is the length of line S_1_–S_2_, which is the transverse horizontal straight-line distance at the widest part of the shoulder. SH is the height of point M, which is the highest point of the shoulder along the line S_1_–S_2_. HW is the length of line H_1_-H_2_, which is the transverse horizontal straight-line distance at the widest part of the hip. HH is the height of point N, which is the highest point of the hip along the line H_1_–H_2_. Each body size data point was measured using a measuring stick (Figure 3b). To match the body size data to each pig, different marks were used to identify different pigs.

### 2.3. Data Collection and Preprocessing

In this study, two types of data were collected: modeling data and test data. The modeling data were used to train the models, and the test data were used as unknown data to test the generalization ability of the trained models. The data collection process in this study was in compliance with European Union legislation concerning the protection of animals for scientific purposes (European Parliament, 2010).

The modeling data were collected in a pig house at the Rongchang Experimental Station, Chongqing, China. There were 50 pens in this pig house and 5 pigs per pen. The area of each pen was 4.2 mL × 2.5 mW × 1.0 mH, and it was equipped with a duckbill drinker. The pig weight and back image data were obtained with the acquisition system. The acquisition system was placed in front of the duckbill drinker. Pigs would enter the system and stand on the weighing platform every time they drank. Therefore, the acquisition system could obtain the back images and body weight (BW) data automatically whenever a pig come to drink throughout the running time of the acquisition system. The body size data of each pig were measured manually once in the morning and once in the afternoon. Each body size data point was measured 5 times. The maximum and minimum were removed and the average of the remaining 3 values was taken as the final result. The result was accurate to within millimeters. Combined with the marks on the pig back, the measured body size data point could be matched to 3D images by the 2D images. The system was cleaned and disinfected every night and put into the following pig pen the next day to start a new collection. Since the pigs had been living in the pig house for some time before the experiment began, they were familiar with the drinking methods, so the pigs were not trained to go to the weighing platform to drink. The data collection period lasted for 88 days. During the experiment, 8 pigs were sold, and 3 pigs died of illness, and a total of 38,112 pig back images and corresponding weight and body size data in various postures from 239 Duroc × Landrace × Yorkshire growing and finishing pigs (121 castrated boars and 129 gilts) were collected (159 images per pig). Pig weight data were in the range of 16.5–117.0 kg, and the number of data points in the weight categories of 16.5–40 kg, >40–65 kg, >65–90 kg, and >90–117 kg was 8094, 10,340, 11,330, and 8348, respectively.

**Figure 4:**
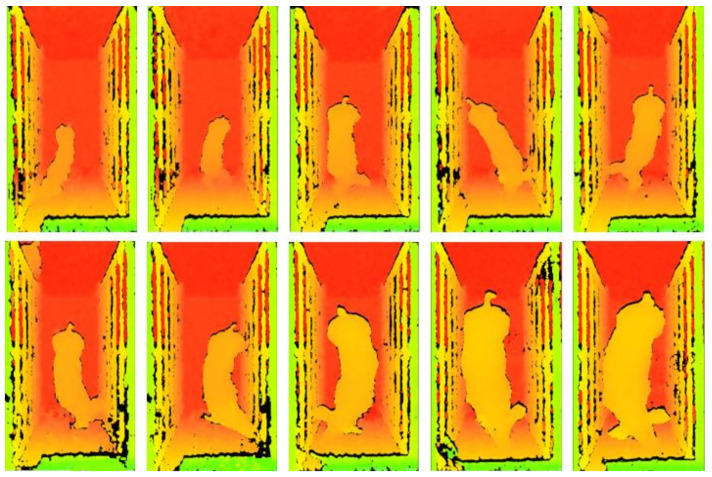
Samples of pig images in various postures.

The test data were collected at a commercial pig farm belonging to the Shandong Rongchang Breeding Company, Binzhou, China. At this farm, pig houses were divided into 10 pens and 20 Duroc × Landrace × Yorkshire growing and finishing pigs were reared in each pen. The area of each pen was 7.5 mL × 4.0 mW × 0.95 mH. The collection method of the test data was the same as that used for the modeling data. The data collection period lasted for 60 days. During the experiment, 4 pigs died of illness and 8 pigs were eliminated, and a total of 20,026 test data in various postures (Figure 4) from 188 pigs in the weight range of 22.0–105.4 kg were collected (106 images per pig). The number of data points in the weight categories of 22.0–42.0 kg, >42.0–62.0 kg, >62.0–82.0 kg, and >82.0–105.0 kg was 6506, 5180, 4154, and 4186, respectively.

**Figure 5:**
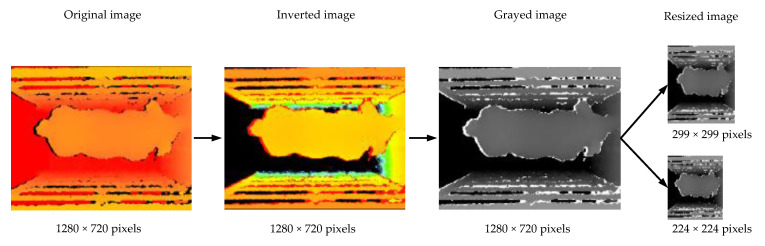
Image preprocessing process.

The size of the original 3D images was 1280 × 720 pixels. To improve training speed, all images were preprocessed in the same way (Figure 5). The distance from the depth camera to the weighing platform was 1650 mm, and the pixel value of each point in the original image was the distance in millimeters from the point to the depth camera. To convert this distance to true height, each pixel value in the images was inverted as

*P*_i_ = 1650 − *P*_o_ (1)

where *P*_i_ represents the pixel’s value in the inverted image, and *P*_o_ represents the pixel’s value in the original image. After inversion, the pixel value of each point was the distance from the point to the weighing platform in the range of 0–1650 mm. Then, the pixel value of the inverted image was scaled into 0–255 and converted into a gray scale image, where the lighter the color, the greater the height. The gray scale image was then resized into 2 different sizes (299 × 299 pixels and 224 × 224 pixels) as the inputs for different models. Since all images were processed in the same way, the process did not change the relative position and size of the pigs in the images, so it had little impact on the final estimation. Finally, each image was tagged with 6 labels in the order of BW, SW, SH, HW, HH, and BL.

### 2.4. Construction, Training and Testing of Pig Weight and Body Size Estimation Models

**Figure 6:**
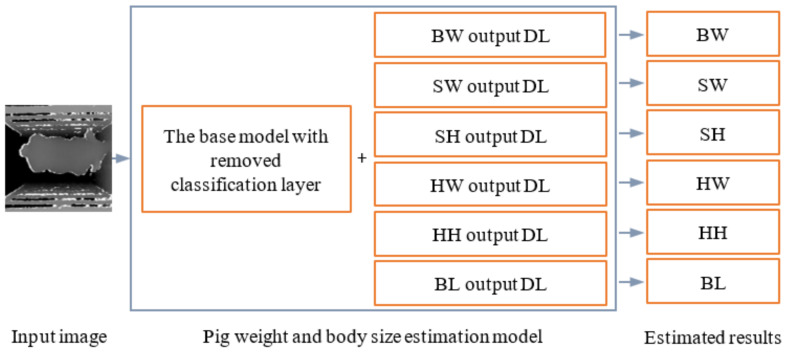
Pig weight and body size estimation model and estimate process. DL: dense layer; BW: body weight; SW: shoulder width; SH: shoulder height; HW: hip width; HH: hip height; BL: body length.

As many CNNs have achieved excellent results in the ImageNet Large Scale Visual Recognition Challenge, 4 state-of-the-art classification CNNs (DenseNet201 [34], ResNet152 V2 [35], Xception [36], and MobileNet V2 [37]) were used as base models and transformed into multiple output regression CNNs for pig weight and body size estimation. DenseNet201 uses dense blocks. In a dense block, the inputs of each layer contain the output of all previous layers and this mechanism can reduce the disappearance of gradients and make the network much deeper. ResNet152 V2 is built based on VGGNet and residual block. The core idea of a residual block is to apply an identity shortcut connection to skip one or more layers directly. This operation can also deepen the network depth. Xception uses depthwise separable convolutions to reduce model size and uses an extreme inception module to fuse features extracted from different convolution kernels. MobileNet V2 is characterized by the use of depthwise separable convolutions and inverted residual structure. The depthwise separable convolution can reduce the parameters of the model and the inverted residual structure can reduce the information loss caused by activation function. The specific transformation process was: (i) the last classification layer of each model was removed; (ii) 6 dense layers (DLs) with only one node and no activation function were added to each model in parallel (Figure 6) to output BW, SW, SH, HW, HH, and BL, separately.

All models were written with the available libraries in Python 3.7.0 and tensorflow-gpu-2.2.0. All code was run on a desktop computer with an Intel i7-9700 processor, 32 GB RAM, Windows 10 (64 bit), and a NVidia GeForce GTX 1660 Ti 6 GB graphics card with Turing^TM^ architecture. The developed computer code was available in GitHub: https://github.com/18801389568/Pig-weight-and-body-size-estimation (accessed on 11 April 2021). Model training is the process of continuously changing model parameters to make the estimation results more accurate. The data used to train the models were modeling data. In all models, the modeling data were randomly divided into training sets and validation sets in a ratio of 7:3 after the order was shuffled. The preprocessed 3D images were used as input during model training, and the output was the corresponding pig weight and body size. As the quantity of data across the weight ranges was similar, the mean square error (MSE) was used as the loss function to evaluate the estimation ability of the models. The MSE was calculated as

(2) $$\mathrm{MSE}={\mathrm{MSE}}_{\mathrm{BW}}+{\mathrm{MSE}}_{\mathrm{SW}}+{\mathrm{MSE}}_{\mathrm{SH}}+{\mathrm{MSE}}_{\mathrm{HW}}+{\mathrm{MSE}}_{\mathrm{HH}}+{\mathrm{MSE}}_{\mathrm{BL}}$$

where MSE_BW_, MSE_SW_, MSE_SH_, MSE_HW_, MSE_HH_, and MSE_BL_ are the MSE generated by estimating BW, SW, SH, HW, HH, and BL, respectively. The calculation methods of MSE_BW_, MSE_SW_, MSE_SH_, MSE_HW_, MSE_HH_, and MSE_BL_ are similar and can be calculated as

(3) $${\mathrm{MSE}}_{V}=\frac{1}{M}\overset{M}{\underset{m=1}{\sum }}{\left(y_{m}^{V}-{\hat{y}}_{m}^{V}\right)}^{2}$$

where V can be any one of BW, SW, SH, HW, HH, and BL; *M* is the total number of data points in the validation set; *m* is the sample number of the data in the validation set; $y_{m}^{V}$ is the measured value for V of the *m*th sample; and ${\hat{y}}_{m}^{V}$ is the estimated result for V of the *m*th sample. In order to compare the performance of each model under the same condition, the configuration of the hyper-parameters used in each model was the same, as shown in Table 1. The loss of each model on the validation set was used as the evaluation standard to retain the best parameters in the training process for each model.

Information about the trained models is shown in Table 2. The number of parameters is the number of all parameters in the model and the number of trainable parameters is the number of parameters except the parameter in batch-normalization layers and global-average-pooling layers. Among the 4 models, the input image size for the modified Xception model is 299 × 299 pixels, while the image size for the other 3 models is 224 × 224 pixels. The model size and number of parameters for modified ResNet152 V2 were largest, while the training time for modified Xception was longest due to the big input image size. Due to modified MobileNet V2 having the smallest model size, the lowest number of parameters, and the smallest input image size, the training time for this model was the shortest.

After model training, test data were used to examine the generalization capability of each model. The models were investigated from the aspect of the estimated root mean square error (RMSE), MAE, mean relative error (MRE), and mean estimation time (MET) of an image.

## 3. Results and Discussion

### 3.1. Model Training Results

**Figure 7:**
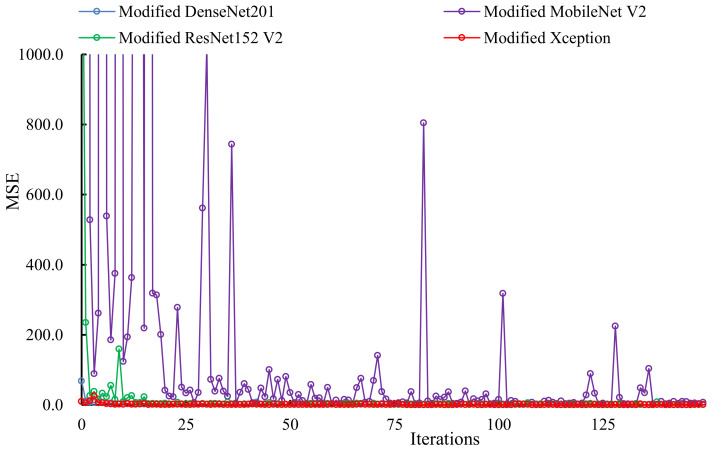
Loss change on validation set of each model.

The change in loss (MSE) of each model on the validation set during the training steps is shown in Figure 7. During the training process, modified MobileNet V2 was observed to experience a larger fluctuation on the validation set. This may be due to the fact that the model has fewer parameters and cannot estimate pig weight and body size well. When the 80th iteration was reached, the other three models had converged and achieved good estimation results. Finally, the lowest MSE obtained by modified DenseNet201, modified MobileNet V2, modified ResNet152 V2, and modified Xception on the validation set were 0.132, 1.243, 0.221, and 0.092, respectively. The modified Xception achieved the highest estimation accuracy.

### 3.2. Model Test Results

Table 3 presents the results of investigating the generalization performance of the models using the test data collected from a commercial pig farm. Similar to the results for the validation set, the four trained models also had good estimation performance. The lowest errors when estimating BW, SW, SH, HW, HH, and BL were obtained by modified Xception, Xception, ResNet152, MobileNet V2, Xception, and ResNet152, respectively. As for the validation set, modified Xception produced the most accurate estimation performance among the four models. This may be because the Xception module in the model can more effectively synthesize information. Although modified MobileNet V2 fluctuated during the training process, it performed well on the test set after training. This is because the task of pig weight and body size estimation is not as complicated as object classification, as it does not need to extract complex textures and edge information from images. When the four models were tasked with estimating body size, the largest MRE was generated by estimating SH. This is because the movement of a pig head when drinking can cause a change in SH. According to observations, when the head of an 80 kg pig moves up and down, it will cause an SH change of about 4 cm. The MET of the four models were all within 27.1 ms, which could meet the requirement of real-time operation.

**Figure 8:**
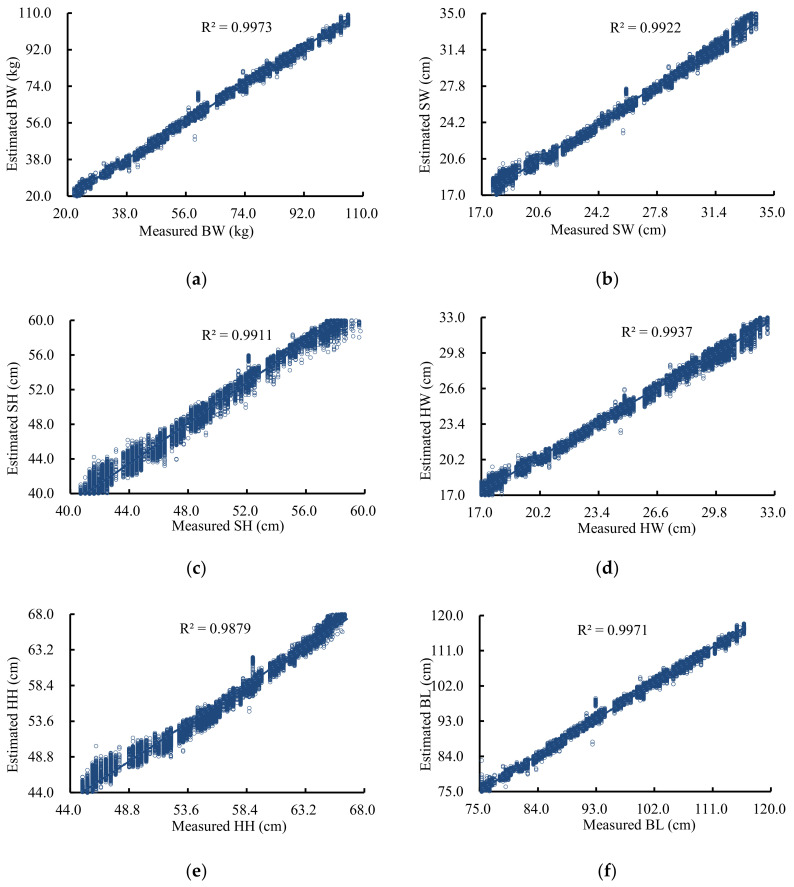
Comparison between measured and estimated BW (**a**), SW (**b**), SH (**c**), HW (**d**), HH € and BL (**f**).

Considering the total MSE of each model, modified Xception was selected as the final pig weight and body size estimation model. Measured and estimated pig weights and body sizes are shown in Figure 8. The coefficient of determination (R^2^) value between the measured and estimated BW, SW, SH, HW, HH, and BL were as high as 0.9973, 0.9922, 0.9911, 0.9937, 0.9879, and 0.9971, respectively. Even if the pig body is not straight, high estimation accuracy can still be obtained. The estimation accuracy of this model is higher than the projection method [14], the 2D image method [15,16,17,18], and the ellipse fitting method [26,27,28,29], as this model estimates pig weight and body size based on the height and distance of all points in a 3D image rather than the individual information points extracted by these other methods. The accuracy is same when using the 3D image method [9,19,20,21,22,23,24,25], but the processing operation of the model is simpler. The estimation accuracy of pig weight and body size cannot be further improved because pig weight changes with eating, drinking, and excretion. Pig weight is also affected by the lean meat ratio. Such changes are difficult to see in images of a pig back, and thus the model cannot tell the difference in pig weight.

### 3.3. Feature Maps

**Figure 9:**
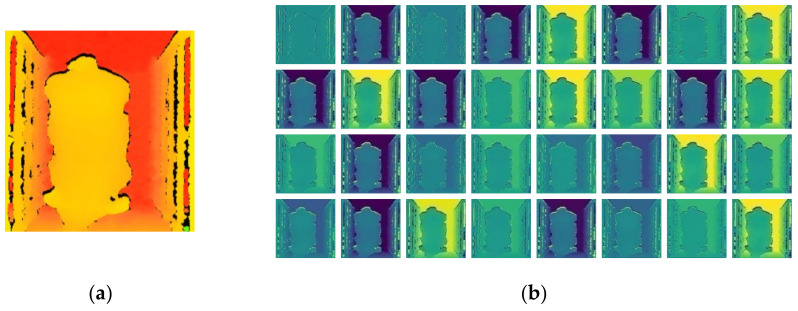
Original image (**a**) and feature maps (**b**) output from the first convolutional layer of modified Xception.

Models can detect elementary features, such as texture and outline, in their shallow convolutional layers and learn to detect more comprehensive features in their deeper layers. To determine what information had been learned and on what basis the modified Xception estimates pig weight and body size, the feature maps that were output by the first convolutional layer were examined. After the original image (Figure 9a) was input to the first convolutional layer of modified Xception, a total of 32 feature maps were output (Figure 9b). When comparing the input image with the feature maps, we found that the input image was smoothed after the first convolutional layer, the background interference was eliminated, the contour, edge, and depth features of the pig body were extracted. Therefore, it was demonstrated that the model estimated pig weight and body size based on a pig height and body shape characteristics. Notably, the model is not necessarily based on the distance between specific points to estimate the body size: it could be based on the overall body physique of the pig, but nevertheless, the performance on the test set showed that the method still produced accurate results. Compared with the method of estimating body size based on the distance of the points in an image, this method might reduce the estimation error caused by posture changes of the pig.

### 3.4. Application Prospect

**Figure 10:**
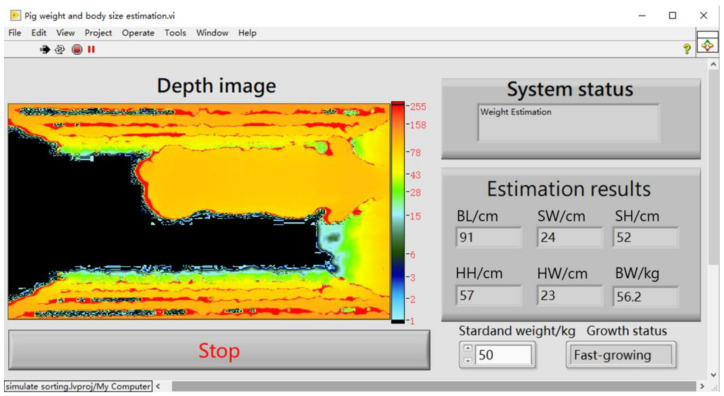
LabVIEW panel of the pig weight and body size estimation system.

Benefitting from the powerful development capability and Python Integration Toolkit provided by LabVIEW, this method can be used to measure pig weight and body size in a fully automated way (Figure 10). Pig weight and body size can be quickly estimated without a complex operation after the preprocessed 3D image was input into the model. Such a simple and convenient operation will reduce the workload and technical requirements for farm breeders. In addition, this non-contact measurement method can also avoid stress or injury to pigs. It is also feasible for the model to be integrated into control programs and be applied to commercial farms.

In conclusion, multiple output regression CNN can be used to accurately estimate pig weight and body size. The estimation process only requires the simple preprocessing of acquired 3D images and can be automated. The high estimation speed of this method can ensure real-time operation in commercial farms. The influence of light on the estimation accuracy can be reduced by using 3D images. Even when we are estimating the weight of pigs of different breeds, only a small amount of pig data need to be collected and corrected on the basis of the original mode output.

## 4. Conclusions

We propose an innovative method of estimating pig weight and body size using a multiple output regression CNN. After training the modified DenseNet201, ResNet152 V2, Xception, and MobileNet V2 on modeling data and comparing the estimation results on test data, modified Xception was finally selected as the optimal pig weight and body size estimation model. This method estimates pig weight and body size based on a pig height, contour, and body shape, and yielded a MAE of 1.16 kg, 0.33 cm, 1.23 cm, 0.38 cm, 0.66 cm, and 0.75 cm when estimating BW, SW, SH, HW, HH, and BL, respectively. The MAT for modified Xception was 0.012 s. This method can successfully estimate pig weight and body size on the LabVIEW platform in a fully automated way. It is feasible to apply this method to sorting systems for fattening pigs, breeding stations and other occasions where there are strict requirements on the speed of pig weight and body size acquisition. This method can also be used to estimate the weight and body size of other animals such as cattle and sheep. Future work should combine modified Xception with object detection technology to realize pig weight and body size estimation through the depth camera installed on the top of the pig house.

## Figures and Tables

**Table 1:** Hyper-parameters of models.

Optimization Function	Learning Rate	Loss Function	Batch Size	Iterations
Adam	0.001	MSE	16	150

**Table 2:** Model information.

Model	Size of Input Image (pixels)	Model Size (MB)	Number of Parameters	Number of Trainable Parameters	Training Time (h)
Modified DenseNet201	224 × 224	229	18,333,510	18,104,454	29.1
Modified MobileNet V2	224 × 224	31	2,265,670	2,231,558	12.9
Modified ResNet152 V2	224 × 224	683	58,343,942	58,200,198	35.7
Modified Xception	299 × 299	243	20,873,774	20,819,246	54.0

**Table 3:** Performance of the models on the test set. BW: body weight; SW: shoulder width; SH: shoulder height; HW: hip width; HH: hip height; BL: body length; RMSE: root mean square error; MAE: mean absolute error; MRE: mean relative error; MET: mean estimation time; MSE: total mean square error.

Items		Modified DenseNet201	Modified MobileNet V2	Modified ResNet152 V2	Modified Xception
BW	RMSE (kg)	2.51	1.84	1.73	1.53
	MAE (kg)	2.03	1.49	1.31	1.16
	MRE	3.44%	2.54%	2.26%	1.99%
SW	RMSE (cm)	0.48	0.44	0.46	0.43
	MAE (cm)	0.38	0.34	0.37	0.33
	MRE	1.49%	1.35%	1.47%	1.31%
SH	RMSE (cm)	1.53	1.38	1.31	1.36
	MAE (cm)	1.42	1.22	1.17	1.23
	MRE	2.79%	2.38%	2.30%	2.40%
HW	RMSE (cm)	0.50	0.40	0.47	0.47
	MAE (cm)	0.45	0.31	0.38	0.38
	MRE	1.84%	1.29%	1.55%	1.58%
HH	RMSE (cm)	1.11	0.96	1.10	0.87
	MAE (cm)	0.90	0.76	0.89	0.66
	MRE	1.59%	1.34%	1.58%	1.16%
BL	RMSE (cm)	1.16	0.89	0.84	0.94
	MAE (cm)	0.97	0.69	0.63	0.75
	MRE	1.05%	0.74%	0.69%	0.82%
MET (ms)		17.98	5.99	27.10	12.32
MSE (kg^2^)		11.699	7.357	7.057	6.236

## Data Availability

Data is contained within the article.
